# Variants in *KCNQ1 *increase type II diabetes susceptibility in South Asians: A study of 3,310 subjects from India and the US

**DOI:** 10.1186/1471-2350-12-18

**Published:** 2011-01-24

**Authors:** Latonya F Been, Sarju Ralhan, Gurpreet S Wander, Narinder K Mehra, JaiRup Singh, John J Mulvihill, Christopher E Aston, Dharambir K Sanghera

**Affiliations:** 1 Department of Pediatrics, College of Medicine, University of Oklahoma Health Sciences Center Oklahoma City, Oklahoma, USA; 2 Hero Dayanand Medical College & Heart Institute, Ludhiana, Punjab, India; 3 All India Institute of Medical Sciences, New Delhi, India; 4 Central University of Punjab, Bathinda, India

## Abstract

**Background:**

Polymorphisms in intron 15 of potassium voltage-gated channel, KQT-like subfamily member 1 (*KCNQ1*) gene have been associated with type II diabetes (T2D) in Japanese genome-wide association studies (GWAS). More recently a meta-analysis of European GWAS has detected a new independent signal associated with T2D in intron 11 of the *KCNQ1 *gene. The purpose of this investigation is to examine the role of these variants with T2D in populations of Asian Indian descent from India and the US.

**Methods:**

We examined the association between four variants in the *KCNQ1 *gene with T2D and related quantitative traits in a total of 3,310 Asian Indian participants from two different cohorts comprising 2,431 individuals of the Punjabi case-control cohort from the Sikh Diabetes Study and 879 migrant Asian Indians living in the US.

**Results:**

Our data confirmed the association of a new signal at the *KCNQ1 *locus (rs231362) with T2D showing an allelic odds ratio (OR) of 1.24 95%CI [1.08-1.43], p = 0.002 in the Punjabi cohort. A moderate association with T2D was also seen for rs2237895 in the Punjabi (OR 1.14; p = 0.036) and combined cohorts (meta-analysis OR 1.14; p = 0.018). Three-site haplotype analysis of rs231362, rs2237892, rs2237895 exhibited considerably stronger evidence of association of the GCC haplotype with T2D showing OR of 1.24 95%CI [1.00-1.53], p = 0.001, permutation p = 8 × 10^-4 ^in combined cohorts. The 'C' risk allele carriers of rs2237895 had significantly reduced measures of HOMA-B in the US cohort (p = 0.008) as well as in combined cohort in meta-analysis (p = 0.009).

**Conclusions:**

Our investigation has confirmed that the variation within the *KCNQ1 *locus confers a significant risk to T2D among Asian Indians. Haplotype analysis further suggested that the T2D risk associated with *KCNQ1 *SNPs may be derived from 'G' allele of rs231362 and 'C' allele of rs2237895 and this appears to be mediated through β cell function.

## Background

The potassium voltage-gated channel, KQT-like subfamily member 1 (*KCNQ1*) is a member of 11 mammalian Kv channel families and has been extensively studied for its role in long QT syndrome. Mutations in *KCNQ1 *have been described to lead to cardiac long QT syndrome, Jervell and Lange-Nielsen syndrome, which are associated with cardiac conduction abnormalities and hearing loss [1]. *KCNQ1 *is expressed mainly in the heart, and to lesser extent in the pancreas, placenta, lung, liver, kidney, brain, and adipose tissue. In addition, *KCNQ1 *is expressed *in vitro *in insulin-secreting cell lines [2]. Insulin secretion from pancreatic β cells is regulated by complex interplay between K_ATP _channels and K_v- _channels and voltage-dependent Ca^++ ^channels [3]. Ionic mechanisms at K_ATP _and K_v- _channels are primarily important in triggering and maintaining glucose-stimulated insulin secretion. However, the contribution of the *KCNQ1 *to the molecular pathogenesis of type II diabetes (T2D) remains to be elucidated.

Recently, two independently conducted genome-wide association studies (GWAS) in Japanese populations have identified *KCNQ1 *as a novel T2D susceptibility gene [4,5]. Intronic variants in the 3' end of *KCNQ1 *(rs2237892, rs2237895, and rs2237897) had a strong association with T2D. Thereafter, association of this locus with T2D was replicated in predominantly East Asian ethnicities including Chinese [6,7], East Asians from Singapore [8], and in some Euro-Caucasians from Denmark [5,9] and Sweden [10]. More recently, a meta-analysis performed on GWAS data from European populations revealed a new independent signal at the *KCNQ1 *locus for another intronic variant (rs231362) associated with T2D at an odds ratio (OR) of 1.08, p = 2.8 × 10^-13 ^[11].

Knowledge of the regulatory role of Kv channels with glucose-stimulated insulin release and recent reports of association of *KCNQ1 *with T2D prompted us to explore the role of these variants in our unique sample from the Punjabi community of India. The validation of GWAS signals in multiple ethnicities is important to putatively define the role of this gene in T2D pathogenesis. A recent replication attempt in three populations from Singapore could not clearly describe the role of these *KCNQ1 *variants for increasing T2D susceptibility in Asian Indians from Singapore because of the small size of their sample [8]. To our knowledge, this is the first study of a population from South Asia reporting the association of two independent GWAS signals in the *KCNQ1 *gene with T2D. Furthermore, this study addresses the possible association of these markers with T2D as one haplotype.

## Methods

### Human Subjects

A total of 3,310 Asian Indians participated in this study from two different cohorts: Group 1 (n = 2,431) is the Punjabi T2D case-control cohort which is part of the Sikh Diabetes Study (SDS) recruited from Northern states of India, including Punjab, Haryana, and Delhi [12]. Group 2 (n = 879) comprises US Asian Indian participants who are first generation immigrants from India and are residents of the states of Oklahoma, Texas, and California. The DNA and serum samples from a total of 1,448 T2D cases and 1,617 normoglycemic (NG) controls and 245 individuals with impaired glucose tolerance (IGT) or impaired fasting glucose (IFG) were studied. The NG control subjects from the Punjabi cohort were random unrelated individuals recruited from the same Asian Indian community as the T2D patients and were matched for ethnicity and geographic location. The US subjects were recruited through public advertisement as part of a population-based study involving free health screening for cardiovascular risk factors. Men and women aged 25-79 years participated. The individuals with mixed ancestry or non-Asian Indian ancestry were not enrolled. Two third of the subjects from the US cohort were originally from the state of Punjab, and the remaining one third were from other western and southern states of India. The diagnoses of T2D were confirmed by reviewing medical records for symptoms, use of medication, and measuring fasting glucose levels following the guidelines of the American Diabetes Association (2004) [13], as described previously [14]. A medical record indicating either (1) a fasting plasma glucose level ≥7.0 mmol/L or ≥126 mg/dL after a minimum 12h fast or (2) a 2h post-glucose level (2h oral glucose tolerance test) ≥11.1 mmol/L or ≥200 mg/dL on more than one occasion, combined with symptoms of diabetes, confirmed the diagnosis. IFG is defined as a fasting blood glucose level ≥100 mg/dL (5.6 mmol/L) but ≤126 mg/dL (7.0 mmol/L). IGT is defined as a 2h OGTT > 140 mg/dL (7.8 mmol/L) but <200 mg/dL (11.1 mmol/L). Subjects with IFG or IGT were considered pre-diabetics and were analyzed separately. The 2h OGTTs were performed following the criteria of the World Health Organizations (WHO) (75 g oral load of glucose). Body mass index (BMI) was calculated as (weight [kg]/height [meter]^2^), and waist-to-hip ratio (WHR) was calculated as the ratio of abdomen or waist circumference to hip circumference. Subjects with type I diabetes, or those having a family member with type I diabetes, or rare forms of T2D sub-types (maturity onset diabetes of young [MODYs]), or secondary diabetes (from e.g. hemochromatosis, pancreatitis) were excluded from the study.

The selection of controls was based on a fasting glycemia <100.8 mg/dL or a 2h glucose <141.0 mg/dL. Subjects with IFG or IGT were excluded when data were analyzed for association of variants with T2D. All blood samples were obtained at the baseline visits. Insulin was measured by radio-immuno assay (Diagnostic Products, Cypress, USA). Homeostasis model assessment for insulin resistance (HOMA-IR) was calculated as (fasting glucose mg/dL × fasting insulin μIU/mL)/405 and for β cell function (HOMA-B) (fasting insulin μIU/mL × 360)/(fasting glucose mg/dL - 63), as described [15]. All participants signed a written informed consent for the investigations. The study was reviewed and approved by the University of Oklahoma Health Sciences Center's Institutional Review Board, as well as the Human Subject Protection Committees at the participating hospitals and institutes in India.

### SNP Genotyping

We genotyped four SNPs from the *KCNQ1 *gene. Three SNPs (rs2237892, rs2237895 and rs2237897) were chosen from intron 15 based on the strong association signals reported in Japanese studies [4,5]. The forth SNP rs231362 from intron 11 was part of a new GWAS signal reported in Caucasian meta-analysis [11]; it maps 150 kb upstream from the Japanese GWAS signal. Genotyping for all four SNPs was performed using TaqMan pre-designed or TaqMan made-to-order SNP genotyping assays from Applied Biosystems Inc. (ABI, Foster City, USA). Genotyping reactions were performed on an ABI 7900 genetic analyzer using 2 uL of genomic DNA (10 ng/uL), following manufacturers' instructions. For quality control, 8-10% replicative controls and 4-8 negative controls were used in each 384 well plate to match the concordance, and the discrepancy rate in duplicate genotyping was <0.2%. Genotyping call rate was 97% or more in all the SNPs studied.

### Statistical Analysis

Data quality for SNP genotyping was checked by establishing reproducibility of control DNA samples. Departure from Hardy-Weinberg equilibrium (HWE) in controls was tested using the Pearson chi-square test. The genotype and allele frequencies in T2D cases were compared to those in control subjects using the chi-square test. Statistical evaluation of genetic effects on T2D risk used multivariate logistic regression analysis with adjustments for age, gender, and other covariates. Continuous traits with skewed sampling distributions (e.g. glucose, insulin, HOMA-IR, and HOMA-B) were log-transformed before statistical analysis. However, for illustrative purposes, values were re-transformed into the original measurement scale. General linear models were used to test the impact of genetic variants on transformed continuous traits (FBG, 2h glucose, HOMA-IR, and HOMA-B) only in NG controls and pre-diabetics + NG controls. Patients were excluded due to confounding effect of glucose lowering medications. Place of birth was used as covariate while analyzing combined sample of the Punjabi and US cohorts. Other significant covariates for each dependent trait were identified by Spearman's correlation and step-wise multiple linear regression with an overall 5% level of significance using SPSS for Windows statistical package (version 18.0) (SPSS Inc., Chicago, USA). Mean values between cases and controls were compared by using an unpaired t-test.

Haplotype analysis of three *KCNQ1 *SNPs was performed using HAPLOVIEW (version 4.0) (http://www.broadinstitute.org/haploview/haploview) which uses an accelerated expectation maximization algorithm to calculate haplotype frequencies. Effect of three-site haplotypes on T2D and quantitative variables were determined using PLINK (version 1.0.6) (http://pngu.mgh.harvard.edu/~purcell/plink/). Meta-analysis was performed by using PLINK for fixed-effects and random-effects models and p value for heterogeneity was derived from Cochrane's Q statistics. To adjust for multiple testing, we used Bonferroni's correction (0.05/number of tests performed).

Statistical power was assessed using the Genetic Power Calculator [16]. The general estimates of power in the Punjabi and combined sample using additive genetic model at α = 0.05, K = 0.18 for detecting the effect sizes between 1.11 (rs231362) and 1.49 (rs2237895) for T2D, were 56% and 89% in the Punjabi and 66% and 97% in combined cohorts, respectively, when the frequency of risk alleles of rs231362 and rs2237895 were 0.73 and 0.42, respectively, in our sample. Our sample lacked power to detect the association of remaining SNPs (rs2237892 and rs2237897) with T2D because these two SNPs were least frequent in our cohorts with the minor allele frequency (MAF) ranging from 1-2%. However, for quantitative traits, the power was well in excess (90%) to detect the inter-genotype difference (e.g. for FBG levels), assuming an additive genetic model, (α = 0.05, and Bonferroni's p = 0.008) at allele frequencies ranging from 0.41-0.98 using 1,048, 569, and 1,617 controls from the Punjabi, US, and combined cohorts, respectively. This power is associated to detect a difference in a quantitative trait of FBG of as little as 1 mg/dL and accounts for an effect size of 0.1 which corresponds to detecting significant βs outside of the range of ± 0.05.

## Results

**Figure 1:**
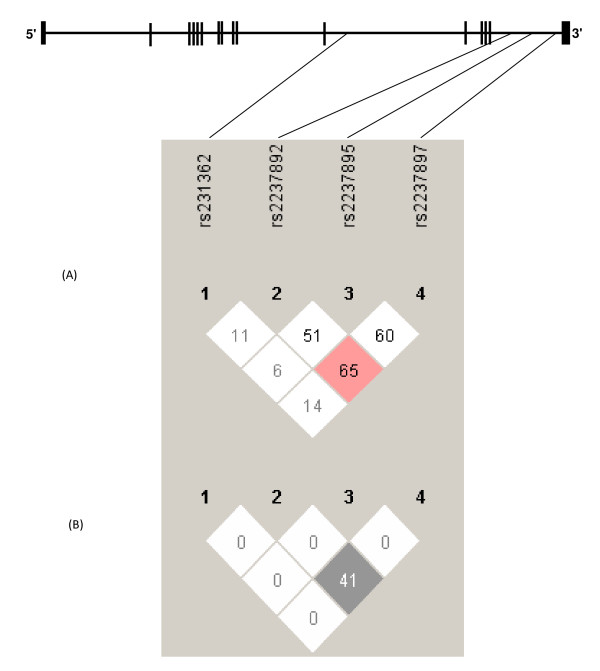
**Figure 1 describes structure of human *KCNQ1 *gene on chromosome 11**. Top portion of the Figure 1 shows the position of four investigated SNPs from intron 11 and 15. Figure 1(A) shows pair-wise linkage disequilibrium (LD) (D') and Figure 1(B) shows pair-wise correlation (r^2^) between SNPs.

Table 1 summarizes and compares the general characteristics of the two study cohorts used in this investigation. Among these subjects, the US cohort was younger and had earlier onset of T2D (42 years) compared to the Punjabi cohort (48 years). Diabetics in the Punjabi cohort had poor glycemic control showing significantly higher FBG levels (by 17 mg/dL) (p = 0.002) compared to the US cohort. HOMA-B (used as a surrogate marker for beta [β] cell function) were also significantly lower (by 33 units) (p = 4.64 × 10^-8^) and WHR significantly higher (by 5 units) (p <0.001) in the Punjabi cohort. The physical location and inter-marker distance of all investigated SNPs is shown in Figure 1. Linkage disequilibrium (LD) analysis revealed that all investigated SNPs were in very modest LD (D' ranging from 0.06-0.65) and showed poor correlation (r^2 ^= 0.00-0.41) with each other. We did not continue genotyping rs2237897 after initial screening of 1,507 samples (815 T2D/692 controls) as this SNP was least frequent in our population; MAF in controls was 1% (additional file 1 Table S1). Compared to all other SNPs, this least frequent SNP was relatively in stronger LD with rs2237892 (D' = 0.65, r^2 ^= 0.41). However, none of the three Japanese GWAS SNPs (rs2237892, rs2237895 and rs2237897) was correlated with rs231362 (r^2^<0.005) (Figure 1).

**Table 1:** Clinical characteristics of study populations (Mean ± SD)

	Punjabi Cohort	US Cohort	Combined
	n = 2,431	n = 879	n = 3,310
Age (yrs.)	53.5 ± 12.9	48.0 ± 13.5	52.1 ± 13.3
% Males	52.5	51.7	52.3
Age at Diagnosis (yrs.)	47.6 ± 11.1	42.4 ± 18.9	47.4 ± 11.6
Duration of Diabetes (yrs.)	7.6 ± 6.8	6.8 ± 7.1	7.6 ± 6.8
BMI (kg/m^2^)	26.9 ± 5.1	26.9 ± 4.5	26.9 ± 5.0
Waist (cm)	93.2 ± 12.4	92.5 ± 13.4	93.0 ± 12.7
WHR	0.95 ± 0.07	0.90 ± 0.13*	0.94 ± 0.10
FBG (mg/dL)			
Non-Diabetic	95.0 ± 13.4	98.5 ± 13.0	96.6 ± 13.3
Diabetic	162.7 ± 62.4	145.9 ± 42.7**	160.8 ± 60.4
2h glucose (mg/dL)			
Non-Diabetic	107.1 ± 23.2	115.4 ± 24.8	111.4 ± 24.4
Diabetic	201.2 ± 69.2	228.2 ± 72.8^¥^	209.1 ± 71.3
Fasting Insulin (IU/mL)	6.6 (6.3 - 6.9)	7.4 (7.1 - 7.8)	6.8 (6.6 - 7.1)
HOMA-IR	2.1 (2.0 - 2.2)	1.9 (1.8 - 2.0)	2.0 (1.9 - 2.1)
HOMA-B	37.9 (35.5 - 40.4)	71.4 (67.5 - 75.5)^φ^	45.7 (43.5 - 48.1)
NG^† ^(%)	43.1	64.7	48.9
T2D^‡ ^(%)	53.8	16.0	43.7
IGT/IFG (%)	3.1	19.2	7.4

^†^Normoglycemic; ^‡^Type II Diabetes; Impaired glucose tolerance, Impaired fasting glucose. *p < 0.001; **p = 0.002; ^¥^p = 0.02 (showing significant difference in the Punjabi and US cohorts); ^φ^p = 4.64 × 10^-8^; Skewed variables (insulin, HOMA-IR and HOMA-B) are presented in mean (95% confidence intervals).			

### Association of KCNQ1 SNPs with T2D

Allelic distribution of all investigated SNPs was in HWE among controls. Two of the three SNPs were associated with T2D in our sample. The strongest association with T2D was seen in rs231362, which remained significant even after adjusting for the effects of age, gender, and BMI. A common 'G' allele conferred a significant risk under additive genetic model in the Punjabi (1.24; 95%CI [1.08-1.43], p = 0.002) and combined cohorts (1.21; 95%CI [1.06-1.37], p = 0.004). Meta-analysis revealed a significant association of this variant in fixed-effects (p = 0.009) but not in random-effects model (p = 0.390), despite the fact that Cochrane's Q statistics did not reveal any significant evidence of heterogeneity between these two data sets (p = 0.114). A moderate association with T2D was also seen in rs2237895 in the Punjabi (1.14; 95%CI [1.01-1.28], p = 0.036) and the combined cohorts (1.15; 95%CI [1.03-1.29], p = 0.011) after adjusting for covariates of age, gender, BMI and place of birth, and this association remained significant in the meta-analysis (1.14, p = 0.018). On the other hand, a non-significant association of rs2237892 with T2D became slightly significant in the meta-analysis both in fixed-effects (p = 0.03) and random-effects models (p = 0.03) (Table 2).

**Table 2:** Genotype distribution and association of *KCNQ1 *SNPs with T2D

Punjabi Cohort (n = 2,355)		US Cohort (n = 710)			Combined (n = 3,065)**					
**SNP**	**Genotype**	**NG**^**† **^**(%)**	**T2D**^**¥ **^**(%)**	Odds Ratios (OR)* (95%CI) *P*-value	**NG (%)**	**T2D (%)**	**Odds Ratios**^**†† **^**(95%CI) *P*-value**	**NG (%)**	**T2D (%)**	**Odds Ratios**^**†† **^**(95%CI) *P*-value**

rs231362	GG	559 (54)	779 (60)	unadjusted 1.25 (1.09 - 1.43)	328 (58)	81 (57)	unadjusted 0.97 (0.72 - 1.29)	887 (56)	860 (60)	unadjusted 1.18 (1.05 - 1.33)
	GA	395 (38)	437 (34)	**0.001**	191 (34)	49 (35)	0.823	586 (37)	486 (34)	**0.005**
	AA	78 (8)	71 (6)	adjusted 1.24 (1.08 - 1.43)	42 (8)	11 (8)	adjusted 1.04 (0.75 - 1.45)	120 (7)	82 (6)	adjusted 1.21 (1.06 - 1.37)
	^**Ψ**^**G**/A	0.73/0.27	0.78/0.22	**0.002**	0.75/0.25	0.75/0.25	0.798	0.74/0.26	0.77/0.23	**0.004**

rs2237892	CC	982 (96.3)	1259 (97.6)	unadjusted 1.49 (0.94 - 2.37)	523 (93.9)	133 (95.7)	unadjusted 1.48 (0.63 - 3.47)	1505 (95.5)	1392 (97.4)	unadjusted 1.74 (1.18 - 2.56)
	CT	36 (3.5)	30 (2.3)	0.094	32 (5.7)	6 (4.3)	0.368	68 (4.3)	36 (2.5)	0.005
	TT	1 (0.1)	1 (0.1)	adjusted 1.44 (0.89 - 2.31)	2 (0.4)	0 (0)	adjusted 2.35 (0.88 - 6.30)	3 (0.2)	1 (0.1)	adjusted 1.53 (1.01 - 2.32)
	**C**/T	0.98/0.02	0.99/0.01	0.136	0.97/0.03	0.98/0.02	0.088	0.98/0.02	0.99/0.01	0.046

rs2237895	AA	359 (35)	410 (32)	unadjusted 1.11 (0.99 - 1.25)	206 (38)	40 (29)	unadjusted 1.32 (1.02 - 1.70)	565 (36)	450 (32)	unadjusted 1.15 (1.04 - 1.27)
	AC	473 (46)	610 (47)	0.068	249 (45)	67 (48)	0.037	722 (46)	677 (48)	0.006
	CC	188 (18)	265 (21)	adjusted 1.14 (1.01-1.28)	96 (17)	32 (23)	adjusted 1.24 (0.93 - 1.66)	284 (18)	297 (20)	adjusted 1.15 (1.03 - 1.29)
	A/**C**	0.58/0.42	0.56/0.44	0.036	0.60/0.40	0.53/0.47	0.146	0.59/0.41	0.55/0.45	0.011

**Meta-analysis**										
SNP	Risk Allele	*P*-value (Fixed Effect)	*P*-value (Random Effect)	OR (Fixed Effect)	OR (Random Effect)	*P*-value (Heterogeneity)				
				
rs231362	G	0.009	0.390	1.15	1.11	0.114				
rs2237892	C	0.030	0.030	1.37	1.37	0.522				
rs2237895	C	0.018	0.021	1.14	1.14	0.313				

* Allelic ORs for Punjabi cohort were adjusted for age, sex, and BMI; ^†^Normoglycemic; ^¥ ^type II diabetes; ^Ψ ^letters in bold indicate risk allele; **Subjects with impaired glucose tolerance (IGT) or impaired fasting glucose (IFG) were excluded from analysis; Bonferroni p-value for three SNPs using 3 genetic models = 0.0055; ^†† ^ORs for US and combined analysis were adjusted for age, gender, BMI, and place of birth.

## Association of KCNQ1 variants with quantitative traits related to obesity and T2D

We investigated the association of *KCNQ1 *variants with quantitative traits associated with obesity and glucose homeostasis including only non-diabetic individuals. Multiple linear regression analysis revealed a moderately significant association of 'A' allele of rs231362 with WHR in the Punjabi cohort (β = -0.02 p = 0.01). A significant decrease in HOMA-B levels, associated with the 'C' risk allele of rs2237895, was observed in the US cohort (p = 0.008) as well as in the meta-analysis both for fixed- effects (p = 0.009) and random-effects (p = 0.009) models (Table 3). This variant also showed a significant association with HOMA-B even in the combined sample including NG and IFG+IGT (β = -0.19; 95%CI [-0.33- -0.06], p = 0.005) (additional file 1 Table S2).

**Table 3:** Multiple regression analysis showing association between SNPs in *KCNQ1 *and obesity and diabetes-related metabolic traits among non-diabetic controls

Punjabi Cohort (n = 1,048)		rs231362		rs2237892		rs2237895				
	**Trait Mean**	**β (95%CI)**	**p adjusted^Ψ^**	**β (95%CI)**	**p adjusted**	**β (95%CI)**	**p adjusted**			

BMI (kg/m^2^)	26.26 (25.94 - 26.58)	0.01 (-0.59 - 0.71)	0.860	-0.05 (-3.03 - 0.46)	0.150	0.04 (-0.23 - 1.13)	0.192			
WHR	0.94 (0.93 - 0.94)	-0.02 (-0.04 - -0.01)	0.010	0.00 (-0.03 - 0.03)	0.992	-0.01 (-0.02 - 0.00)	0.149			
FBG (mg/dL)	95.18 (94.30 - 96.05)	-0.04 (-0.02 - 0.01)	0.220	-0.06 (-0.08 - 0.01)	0.088	-0.01 (-0.03 - 0.01)	0.310			
INSULIN (IU/mL)	7.35 (6.82 - 7.92)	0.06 (-0.03 - 0.28)	0.107	0.03 (-0.25 - 0.52)	0.496	-0.06 (-0.21 - 0.18)	0.870			
HOMA-IR	1.66 (1.54 - 1.80)	-0.04 (-0.46 - 0.15)	0.309	0.02 (-0.30 - 0.49)	0.634	-0.11 (-0.32 - 0.93)	0.282			
HOMA-B	85.09 (77.74 - 93.14)	0.05 (-0.06 - 0.27)	0.223	0.07 (-0.08 - 0.81)	0.106	-0.07 (-0.43 - 0.03)	0.091			

**US Cohort (n = 569)**		rs231362		rs2237892		rs2237895				

	Trait Mean	β (95%CI)	p adjusted^Ψ^	β (95%CI)	p adjusted	β (95%CI)	p adjusted			

BMI (kg/m^2^)	26.19 (25.83 - 26.55)	0.07 (-.030 - 2.43)	0.124	0.09 (0.11 - 2.90)	0.034	0.07 (-0.08 - 1.34)	0.082			
WHR	0.88 (0.87 - 0.89)	-0.02 (-0.04 - 0.00)	0.197	-0.04 (-0.26 - 0.10)	0.395	-0.04 (-0.05 - 0.01)	0.289			
FBG (mg/dL)	95.95 (95.05 - 96.85)	-0.01 (-0.03 - 0.00)	0.086	0.04 (-0.02 - 0.06)	0.373	0.03 (-0.02 - 0.03)	0.536			
INSULIN (IU/mL)	6.72 (6.37 - 7.09)	0.04 (-0.05 - 0.12)	0.393	0.01 (-0.99 - 1.19)	0.862	-0.09 (-0.15 - -0.05)	0.037			
HOMA-IR	1.57 (1.49 - 1.66)	0.02 (-0.17 - 0.25)	0.704	0.02 (-0.20 - 0.34)	0.622	-0.11 (-0.30 - -0.03)	0.015			
HOMA-B	79.71 (74.87 - 84.86)	0.09 (-0.01 - 0.25)	0.062	-0.03 (-0.43 - 0.20)	0.490	-0.13 (-0.37 - -0.06)	**0.008**			

**Meta-analysis (n = 1,617)**		rs231362			rs2237892			rs2237895		

	Trait Mean	P(F)^1^	P(R)^2^	P(H)^3^	P(F)	P(R)	P(H)	P(F)	P(R)	P(H)

BMI (kg/m^2^)	26.23 (25.99 - 26.48)	0.856	0.849	0.051	0.640	0.990	0.010	0.463	0.463	0.670
WHR	0.91 (0.91 - 0.92)	0.840	0.840	0.445	0.396	0.396	0.932	0.118	0.118	0.399
FBG (mg/dL)	95.48 (94.84 - 96.12)	0.020	0.020	0.551	0.555	0.553	0.284	0.596	0.725	0.133
INSULIN (IU/mL)	7.09 (6.75 - 7.46)	0.467	0.653	0.032	0.012	0.488	0.003	0.248	0.248	0.539
HOMA-IR	1.62 (1.54 - 1.71)	0.689	0.689	0.738	0.078	0.630	0.001	0.616	0.616	0.680
HOMA-B	82.71 (78.06 - 87.63)	0.062	0.062	0.912	0.475	0.766	0.293	**0.009**	**0.009**	0.532

^Ψ^*P *values were adjusted for age, gender and BMI in the Punjabi cohort; age, gender, BMI and place of birth in US cohorts; Bonferroni p = 0.008; ^1^p-value for fixed effect; ^2^p-value for random effect; ^3 ^p-value for heterogeneity; measures on serum insulin, HOMA-IR, and HOMA-B were available in 657 of 1,048 control individuals in the Punjabi cohort.										

## Haplotype analysis

To determine whether these SNPs demonstrate any additional evidence of association with T2D when examined together, we performed a haplotype analysis. As shown in Table 4, the three-site haplotype analysis using rs231362, rs2237892 and rs2237895 revealed a significant difference in the distribution of haplotype among cases and controls in both the Punjabi and combined cohorts showing significant global χ^2 ^(χ_3_^2 ^= 12.9, p = 0.005 and χ_3_^2 ^= 14.1, p = 0.003), respectively. The least frequent variant (rs2237897) was not included in haplotype analysis. The second most frequent haplotype (GCC) was significantly more prevalent among T2D cases (0.34) compared to NG controls (0.30) in both the Punjabi (p = 0.002) as well as combined cohorts (p = 4.07 × 10^-4^). Association of the GCC haplotype with T2D remained significant after adjusting for age, gender, and BMI in the Punjabi cohort (1.31 95%CI [1.04-1.65], p = 0.004 [adjusted], p = 0.006 [permutation]), and adjusting for age, gender, BMI, and place of birth in combined cohorts (1.24 95%CI [1.00-1.53], p = 0.001 [adjusted], p = 8 × 10^-4 ^[permutation]) (Table 4).

**Table 4:** Estimated haplotype probabilities and χ2- test showing haplotype association between *KCNQ1 *SNPs (rs231362, rs2237892, rs2237895) and T2D

	Punjabi Cohort						
**Haplotype**	**NG**	**T2D**	**χ^2^**	**Adjusted OR (95%CI)**	**Unadjusted p**	**Adjusted p**	**Permutation p (5000)**

ACC	0.12	0.11	1.94	0.86 (0.70 -1.05)	0.145	0.184	0.489
**GCC**	0.30	0.34	7.65	1.22 (1.07 -1.39)	**0.002**	**0.004**	**0.006**
**ACA**	0.14	0.12	7.39	0.74 (0.61-0.90)	**0.003**	**0.007**	**0.019**
GCA	0.44	0.44	0.02	1.02 (0.91-1.15)	0.743	0.836	0.997
	global χ^2 ^= 12.9, df = 3, p = 0.005						

	US Cohort						

Haplotype	NG	T2D	χ^2^	Adjusted OR (95%CI)	Unadjusted p	Adjusted p	Permutation p (5000)

ACC	0.10	0.13	1.61	1.67 (1.03 - 2.72)	0.142	0.135	0.507
GCC	0.30	0.35	2.58	1.22 (0.90 - 1.65 )	0.137	0.101	0.496
ACA	0.14	0.11	1.53	0.72 (0.44 - 1.17)	0.350	0.233	0.878
GCA	0.46	0.41	2.06	0.88 (0.66 - 1.19)	0.176	0.197	0.575
	global χ^2 ^= 7.05, df = 3, p = 0.070						

	Combined (Punjabi + US Cohorts)						

Haplotype	NG	T2D	χ^2^	Adjusted OR (95%CI)	Unadjusted p	Adjusted p	Permutation p (5000)

ACC	0.12	0.11	0.31	0.96 (0.80 -1.15)	0.644	0.611	0.985
**GCC**	0.30	0.34	10.1	1.22 (1.09 -1.36)	**4.07 × 10**^**-4**^	**0.001**	**8.00 × 10**^**-4**^
**ACA**	0.14	0.12	8.37	0.77 (0.64 -0.91)	**0.002**	**0.002**	**0.011**
GCA	0.44	0.44	0.45	0.98 (0.88 -1.09)	0.734	0.826	0.991
	global χ^2 ^= 14.1, df = 3, p = 0.003						

Analysis were adjusted for age, gender and BMI in the Punjabi cohort; age, gender, BMI, and place of birth in US and combined (Punjabi + US) cohorts

## Discussion

Three variants (rs2237892, rs2237895, and rs2237897) from intron 15 of the *KCNQ1 *gene were the SNPs most frequently associated with T2D and fasting insulin levels in previous studies on East Asian populations [4,5,8]. Our data could not replicate the association of rs2237892 or rs2237897 with T2D or fasting serum insulin levels in our Asian Indian sample and these findings are in agreement with the absence of association with these variants seen in other Caucasian studies [17-19]. Perhaps a significant ethnic difference in the allelic distribution could be the reason of non-replication as the MAF of these SNPs was significantly lower in our sample compared to East Asian populations: 2-3% vs. 28-41%, respectively, for rs2237892 and 1% vs. 28-39%, respectively, for rs2237897 [4-6,8]. Given the low allele frequency of rs2237892 and rs2237897 in Asian Indians, the statistical power of our study to identify any association with T2D or related metabolic traits is low, which may explain the lack of association with these SNPs in our sample. Moreover, our data revealed a significant variation in LD patterns in these SNPs compared to East Asians; for instance, there was a strong LD between rs2237892, rs2237895 and rs2237897 SNPs in East Asians (D' = 0.84-0.98, r^2 ^= 0.20-0.66) [7] compared to our Asian Indian cohort (D' = 0.51-0.65, r^2 ^= 0.0-0.41). These differences can also increase or decrease the disease risk; best example for this is the negative association of *TCF7L2 *SNPs with T2D in Chinese [20]. Nevertheless, despite those differences, our data replicated a strong association of a new GWAS signal at the *KCNQ1 *locus (rs231362) with T2D (1.24, p = 0.002) in our Punjabi cohort, identified recently in Caucasian meta-analysis [11]. This association appears to be independent of the other SNPs (rs2237892, rs2237895 and rs2237897) as these 3'SNPs were poorly correlated with rs231362 (D' = 0.06-0.14, r^2 ^= 0.00) in our sample. A similar poor correlation of these SNPs with rs231362 was observed in the Caucasian meta-analysis (r^2 ^= 0.05) [11]. It is possible that the observed association of rs231362 with T2D could be mediated by obesity, as the same T2D protective 'A' allele is linked with low WHR among NG controls in the Punjabi cohort (p = 0.01). However, after controlling for the effect of WHR, the significance of this association with T2D did not disappear (p = 0.003), suggesting that the association of this SNP with T2D could be independent of the pathway related to obesity. Additionally, our data also showed a moderate association of the rs2237895 variant with T2D in the Punjabi (OR 1.14, p = 0.036) and in meta-analysis (OR 1.14, p = 0.02). However, after applying Bonferroni's correction for multiple testing, these associations did not remain statistically significant. On the other hand, the same variant (rs2237895) revealed a strong association with HOMA-B in both the US (adjusted p = 0.008; Bonferroni p = 0.008) as well as in meta-analysis (p = 0.009), which may increase the risk of insulin resistance and T2D susceptibility in this population. A non-significant trend associated with HOMA-B in the same direction was also observed our Punjabi cohort. The measures on fasting insulin were only available on 63% of NG controls from the Punjabi cohort which also could have contributed to less significant association with HOMA-B in this cohort. In addition, the Punjabi cohort had poor β cell function as indicated by their significantly lower HOMA-B levels compared to the US cohort (p = 4.64 × 10^-8^) (Table 1). Perhaps, relatively younger mean age (5.5 years) of the US cohort, sample heterogeneity, and/or migration might be contributing for this difference that has resulted in genotype difference in affecting HOMA-B levels.

The role of these *KCNQ1 *variants for increasing T2D susceptibility is further substantiated when we tested these three variants together (rs231362, rs2237892, rs2237895) using haplotype analysis. The at-risk GCC haplotype revealed a strong association with T2D and conversely, the ACA haplotype revealed a significant protection in both Punjabi and combined cohorts and a non-significant trend was also noticed in US cohort. Notably, in haplotype analysis, it appears that the 'G' allele of rs231362 contributes to T2D risk only in the presence of 'C' allele of rs2237895 on GCC haplotype and not when it is on GCA haplotype. These findings suggest that the observed association of GCC haplotype of with T2D may be derived from rs231362 and rs2237895. Interestingly, the increased T2D risk associated with the GCC haplotype and decreased T2D risk associated with ACA haplotype remained unchanged after including HOMA-IR along with other covariates (age and gender) in the model showing ORs of (1.21; 95%CI [1.08-1.37], p = 0.001) for GCC and (0.77; 95%CI [0.64-0.92], p = 0.004) for ACA. These results further suggest that the *KCNQ1 *risk with T2D may be mediated through β cell function rather insulin resistance. To further understand the mechanism of association of this GCC haplotype with T2D, we investigated whether these haplotypes have any role in affecting T2D-related quantitative phenotypes. Our data could not verify association of any of these haplotypes with quantitative phenotypes related to obesity (BMI, waist and WHR) and glucose homeostasis (FBG, 2h glucose, HOMA-IR and HOMA-B) (data not presented).

Although, these results have independently replicated a strong association of rs231362 with T2D and also a strong association of the GCC halpotype with T2D in this Asian Indian sample, these data provided little guidance in determining the putative role of these variants (individually or in haplotype combination) in the biology of T2D. Even the variant showing strongest association with T2D in Punjabi cohort in single SNP analysis (rs231362) was weakly correlated with FBG (p = 0.02) and HOMA-B (p = 0.062) in meta-analysis (Table 3). These findings are in agreement with the previous Caucasian studies, and suggest the possibility of different mechanisms controlling normal glucose homeostasis and the development of T2D [11]. On the other hand, our results showed a strong association of rs2237895 with HOMA-B indicating that the NG individuals who carried the at-risk 'C' allele also had reduced measures of fasting serum insulin (p = 0.037) and significantly reduced HOMA-B levels in the US cohort (p = 0.008) and also was revealed a strong association with HOMA-B in meta-analysis (p = 0.009). A similar association of rs2237895 with lower insulin secretion and impaired β cell function was reported in other study performed in Danish [9] and Scandinavian populations [10]. Incidentally, the MAF of this variant in Danes (42.5%) was also similar to our Asian Indian sample (41%). Also, consistent with our study, no other *KCNQ1 *variant was associated with insulin secretion or β cell function in Danes. The small size of our US cohort is a limitation of our study as the observed association of rs231362 with T2D in our primary (Punjabi) cohort could not be confirmed in the US cohort due to small size and/or cohort heterogeneity, although the cohort heterogeneity could not confirmed through Cochrane statistics in meta-analysis (p[heterogeneity] = 0.114), perhaps, because only two datasets were meta-analyzed (Table 2). Also, the MAF in all these SNPs did not vary significantly among NG individuals of the Punjabi and US cohort (0.27 vs. 0.25 for rs231362, 0.02 vs. 0.03 in rs2237892, and 0.42 vs. 0.40 in rs2237895), respectively. Therefore, future confirmation of these results, including association of haplotypes on larger cohorts from South Asia will help verify the association of *KCNQ1 *with T2D in the populations of Indian sub-continent.

## Conclusions

Our study has replicated a previously reported association of rs231362 with T2D in a non-Caucasian and non- East Asian sample from Punjab, India. Our haplotype data have further revealed a strong association of the GCC haplotype with T2D in this sample. Our results have also independently confirmed the association of rs2237895 with HOMA-B, which may be linked to β cell dysfunction. However, as the overall risk explained by these SNPs for susceptibility to T2D or β cell dysfunction is <1.3, further functional studies are warranted to clearly delineate the role of the *KCNQ1 *locus in normal glucose homeostasis and T2D pathogenesis. Perhaps these SNPs are good proxies for the yet undetected causal SNP which could be part of this gene or nearby genes. Extensive dense genotyping and resequencing in this region should identify causative variants in the *KCNQ1 *locus explaining its functional association with insulin secretion and T2D.

## Abbreviations

*KCNQ1*: The potassium voltage-gated channel, KQT-like subfamily member 1; T2D: Type II diabetes; GWAS: Genome-wide association study; HOMA-IR: Homeostasis model assessment for insulin resistance; HOMA-B: Homeostasis model assessment for β cell function; SNP: Single nucleotide polymorphism; FBG: Fasting blood glucose; BMI: Body mass index; WHR: Waist-to-hip ratio; MAF: Minor allele frequency.

## Competing interests

The authors declare that they have no competing interests.

## Authors' contributions

All authors have read and approved the final manuscript. LB carried out the molecular genetic studies and carried out statistical analysis, SR, GSW, NKM and JS participated in recruitment of study subjects, study design and manuscript editing. JJM helped the subject recruitment from the US and manuscript editing. CEA helped in statistical analysis. DKS is principal investigator and coordinator of the project and was involved in conceptualizing the project, study design, genotyping data quality control, analysis, interpretation, manuscript drafting and finalizing the manuscript.

## Pre-publication history

The pre-publication history for this paper can be accessed here:

http://www.biomedcentral.com/1471-2350/12/18/prepub

## Supplementary Material

Additional file 1 **Table S1 and S2**. Click here for file
