# Supplementary material for: Variants in KCNQ1 increase type II diabetes susceptibility in South Asians: A study of 3,310 subjects from India and the US
Source: BMC Med Genet. 2011 Jan 24;12:18. doi: 10.1186/1471-2350-12-18 (PMC3037841; doi:10.1186/1471-2350-12-18)
Supplement: Additional file 1 — Table S1 and S2. [file 1471-2350-12-18-S1.DOC]

Additional Files

| Table S1. Genotype distribution and association of  *KCNQ1* SNP (rs2237897) with type 2 diabetes | | | | | | | | | | |
| --- | --- | --- | --- | --- | --- | --- | --- | --- | --- | --- |
|  |  | Punjabi Cohort (n=1,507) | | | US Cohort (n=710) | | | Combined (n=3,065) | | |
| SNP | Genotype | NG^†^ (%) | T2D^¥^  (%) | Odds Ratios (OR)* (95%CI)  *P* value | NG (%) | T2D (%) | Odds Ratios (OR)* (95%CI)  *P* value | NG (%) | T2D (%) | Odds Ratios (OR)* (95%CI)  *P* value |
| rs2237897 | CC | 672 (97) | 803 (99) |  | -- | -- |  | -- | -- |  |
|  | CT | 20 (3) | 12 (1) |  | -- | -- |  | -- | -- |  |
|  | TT | 0 | 0 |  | -- | -- |  | -- | -- |  |
|  | C/T | 0.99/0.01 | 0.99/0.01 | 0.51 (0.25 - 1.07), p=0.176** | -- | -- | -- | -- | -- | -- |
| *ORs were adjusted for age, sex, and BMI; ^†^normoglycemic; ^¥^ type II diabetes; **subjects with impaired glucose tolerance were excluded from analysis. | | | | | | | | | | |

| Table S2. Association between SNPs in *KCNQ1* and obesity and diabetes-related metabolic traits among non-diabetics and individuals with IGT and IFG | | | | | | | | |
| --- | --- | --- | --- | --- | --- | --- | --- | --- |
| Punjabi Cohort ( N=1,124) | | rs231362 | | rs2237892 | | rs2237895 | | |
|  | Trait Mean | β (95%CI) | p adjusted^Ψ^ | β (95%CI) | p adjusted | β (95%CI) | p adjusted | |
| BMI (kg/m^2^) | 26.36 (26.05 - 26.67) | 0.00 (-0.59 - 0.65) | 0.921 | -0.04 (-3.00 - 0.45) | 0.147 | 0.04 (-0.25 - 1.06) | 0.222 | |
| WHR | 0.94 (0.93 - 0.94) | -0.02 (-0.04 - 0.00) | 0.023 | -0.01 (-0.03 - 0.02) | 0.796 | -0.04 (-0.02 - 0.00) | 0.174 | |
| FBG (mg/dL) | 95.04 (94.15 - 95.93) | -0.04 (-0.03 - 0.01) | 0.230 | -0.05 (-0.08 - 0.01) | 0.124 | -0.03 (-0.03 - 0.01) | 0.462 | |
| INSULIN (IU/mL) | 7.49 (6.97 - 8.05) | 0.05 (-0.05 - 0.24) | 0.198 | 0.03 (-0.20 - 0.56) | 0.362 | -0.02 (-0.24 - 0.13) | 0.571 | |
| HOMA-IR | 1.72 (1.59 - 1.85) | -0.04 (-0.43 - 0.16) | 0.381 | 0.03 (-0.24 - 0.53) | 0.467 | -0.05 (-0.33 - 0.07) | 0.217 | |
| HOMA-B | 84.41 (77.40 - 92.06) | -0.04 (-0.47 - 0.19) | 0.402 | 0.07 (-0.04 - 0.82) | 0.076 | -0.08 (-0.45 - 0.00) | 0.054 | |
| US Cohort (N= 738) | | rs231362 | | rs2237892 | | rs2237895 | | |
|  | Trait Mean | β (95%CI) | p adjusted | β (95%CI) | p adjusted | β (95%CI) | | p adjusted |
| BMI (kg/m^2^) | 26.62 (26.30 - 26.95) | 0.04 (-0.20 - 0.83) | 0.226 | 0.09 (0.32 - 3.12) | 0.016 | 0.07 (-0.07 - 1.22) | | 0.078 |
| WHR | 0.88 (0.88 - 0.89) | -0.01 (-0.03 - 0.00) | 0.127 | -0.05 (-0.24 - 0.04) | 0.150 | -0.04 (-0.04 - 0.01) | | 0.231 |
| FBG (mg/dL) | 98.46 (97.50 - 99.44) | -0.05 (-0.06 - 0.01) | 0.189 | -0.01 (-0.05 - 0.04) | 0.802 | -0.05 (-0.03 - 0.01) | | 0.149 |
| INSULIN (IU/mL) | 7.14 (6.80 - 7.49) | 0.03 (-0.06 - 0.14) | 0.413 | 0.03 (-0.49 - 1.08) | 0.459 | -0.10 (-0.28 - -0.04) | | 0.011 |
| HOMA-IR | 1.72 (1.63 - 1.81) | 0.05 (-0.05 - 0.16) | 0.284 | 0.06 (-0.09 - 0.44) | 0.188 | -0.10 (-0.29 - -0.02) | | 0.021 |
| HOMA-B | 78.28 (74.06 - 82.74) | 0.08 (-0.01 - 0.22) | 0.071 | 0.01 (-0.79 - 1.03) | 0.801 | -0.17 (-0.31 - -0.03) | | 0.021 |
| Combined (N= 1,862) | | rs231362 | | rs2237892 | | rs2237895 | | |
|  | Trait Mean | β (95%CI) | p adjusted | β (95%CI) | p adjusted | β (95%CI) | | p adjusted |
| BMI (kg/m^2^) | 26.46 (26.24 - 26.69) | 0.02 (-0.28 - 0.63) | 0.456 | 0.02 (-0.61 - 1.66) | 0.362 | 0.04 (-0.07 - 0.88) | | 0.091 |
| WHR | 0.92 (0.91 - 0.92) | -0.04 (-0.04 - 0.00) | 0.063 | -0.04 (-0.20 - 0.02) | 0.115 | -0.01 (-0.01 - 0.00) | | 0.164 |
| FBG (mg/dL) | 96.55 (95.89 - 97.21) | -0.03 (-0.02 - 0.00) | 0.173 | -0.02 (-0.04 - 0.02) | 0.370 | -0.01 (-0.03 - 0.00) | | 0.102 |
| INSULIN (IU/mL) | 7.34 (7.01 - 7.68) | 0.05 (-0.01 - 0.18) | 0.076 | 0.04 (-0.08 - 0.39) | 0.202 | -0.05 (-0.22 - 0.02) | | 0.101 |
| HOMA-IR | 1.72 (1.64 - 1.80) | 0.03 (-0.06 - 0.14) | 0.411 | 0.04 (-0.08 - 0.40) | 0.192 | -0.07 (-0.26 - -0.02) | | 0.024 |
| HOMA-B | 81.44 (77.27 - 85.83) | 0.04 (-0.03 - 0.18) | 0.163 | 0.05 (-0.05 - 0.48) | 0.106 | -0.19 (-0.33 - -0.06) | | 0.005 |
| ^Ψ^*P* values were adjusted for age, gender and BMI in the Punjabi cohort; age, gender, BMI and place of birth in US and combined cohorts; Bonferroni p ≤ 0.008 | | | | | | | | |
